# Which Parenting Style Encourages Healthy Lifestyles in Teenage Children? Proposal for a Model of Integrative Parenting Styles

**DOI:** 10.3390/ijerph16112057

**Published:** 2019-06-11

**Authors:** Paloma Alonso-Stuyck

**Affiliations:** Àrea de Psicologia i Salut Mental, Institut d’Estudis Superiors de la Família, Universitat Internacional de Catalunya, 08017 Barcelona, Spain; palonsos@uic.es

**Keywords:** model of integrative parenting styles, healthy lifestyles, teenage years

## Abstract

Given that we live in an environment in constant change—a liquid society, according to Bauman—we propose a versatile parenting style with the capacity to adapt to the variations of socio-temporal evolution. This is achieved by basing parenting guidelines on the permanent, executive, cognitive, and affective components of a person. Although the first reviews of parenting styles emphasized the Authoritative style, the emerging tendency in some geographical areas has been to prioritize the Indulgent style. Extracting the common factor of these two styles, the suggestion is to improve the affective aspect of the relationship characterized by warm and close parent–child interactions. It is important to respect the style of each family in order to support it in its educational task while offering guidelines to help consolidate healthy adolescent lifestyles. In this line, we present successful experiments that have helped families in this decisive task by highlighting the efficacy of promoting systemic educational plans that involve the whole society.

## 1. Introduction

Today, adolescence is conceived as being a turning point in the life cycle, a stage when a person starts not only to make personal decisions, but also to develop an identity for his/herself. It is well-known that many healthy behaviors co-vary during adolescence and establish lifestyles [1]. Thus, this period is an opportunity to consolidate a healthy lifestyle. As G. Stanley Hall, the first president of the American Psychological Association, pointed out, a new birth of more complex and profound human traits takes place during adolescence. This study considers the concept of the Healthy Lifestyle as a general way of life resulting from the interaction between life conditions in a broad sense and individual behavior patterns resulting from personal and sociocultural characteristics [2]. We also recognize the tridimensional classification of Healthy Lifestyles proposed by Desarrollo Humano Integral, DHi, which includes [3] (a) Daily Life Skills: sleep, eating, hydration, hygiene and skincare, physical activity; (b) Proactivity: emotional, financial, environmental, and at home; and (c) Time Management: work/family, rest, and addiction prevention.

This conceptual base is used to interpret the results of an intercultural study on healthy lifestyles and their relationship with parenting styles across different latitudes [4,5,6,7,8,9,10,11,12]. In these studies, healthy adolescent lifestyles are often associated with family child-rearing styles, as both stem from health areas that dictate responsibility for one’s own personal wellbeing [13] and from the psycho-pedagogical perspective focused on personal development [14]. Both traditions indicate that family atmosphere influences self-regulation capacity, which is indispensable for resisting environmental and media pressures toward adolescent risk behaviors [3]. More specifically, the most important factor is affective dimension—or responsiveness, care, and respect for the uniqueness of the child.

An integrative definition of Parenting Styles would include a set of parental attitudes, feelings, and behavior patterns of parents toward their children that influence their psychological and social functioning [15]. Darling and Steinberg [16], reviewing the data provided by the study of the two traditions mentioned (parenting styles and practices), understand parenting styles as the context where specific child-rearing guidelines exercise their influence. Thus, their definition incorporates the ecological theory of human development, whereby an adolescent’s identity is revealed through the interaction between his/her individual life cycle [17] and his/her ecological niche (family and sociocultural contexts) [18].

If, in addition to the previous multi-contextual considerations, we include the time coordinate to the received models, we can deduce that the intergenerational transmission of family educational styles is an unconscious process [19], and that its effects last throughout a life cycle and affect personal development [9,20,21]. Therefore, when advising families about how to improve their parenting styles, it would first be necessary to help them to become aware of these received models. Once they recognize these models, it becomes possible to offer respectful alternatives to the identity of each family that would allow it to adapt to the dynamic changes of generational sensitivity.

This study aims to provide an integrated synthesis of the two traditions mentioned—pedagogical and therapeutic—and situate them within the permanent and psychological dimensions of a person. Indeed, situating the educational guidelines in the three components of personality—executive, cognitive, and affective [22]—means giving them vital plasticity and rendering them adaptable to the changes stemming from the passing of time and from cultural characteristics, thus offering temporal and multi-contextual flexibility [23]. This would enable each family to introduce variations in its constitutive essence—such as reciprocal and loving relationships between genders and generations—[24] and to consider the distinct genetic sensitivity and environmental susceptibility of each child [8,25] without renouncing its own family style. This flexibility would play a mediator role in the wellbeing of its members and in the prevention of new social diseases, thus guiding the way to dealing with the paradoxes of our liquid society [26], the consequences of globalization, and postmodernism [27]. As the preventative cornerstone of risky adolescent lifestyles, this decisive mediator role requires both a recognition of the family as an invention unit and the complete development of the adolescent’s citizenship [28].

## 2. Proposal for a Versatile Triangle of Parenting Styles

Diana Baumrind investigates parenting styles by drawing on Lewin’s leadership styles—authoritarian, democratic, and laissez-faire—which describe different ways to exert power in groups. She defined parental educational styles as relationship patterns that typify the interactions between parents and children. She added an intermediate style to Lewin’s proposals— Authoritative—which involves exercising authority by taking the child’s opinions into account. Completing her proposal, Maccoby and Martin [29] crossed the two variables on an axis of the following coordinates: Control—to fulfill norms and establish limits, and Responsiveness—warm acceptance of the child’s opinion. Thus were established the four traditional parenting styles, which are as follows: Authoritative (characterized by responsiveness and high control), Indulgent (low control and high responsiveness), Authoritarian (high control and low responsiveness), and Neglectful (low control and low responsiveness). Their typology combined a behavioral variable, control, inserted in the executive component of the personality, with an affective component, responsiveness, rooted in the emotional dimension of the personality.

**Figure 1:**
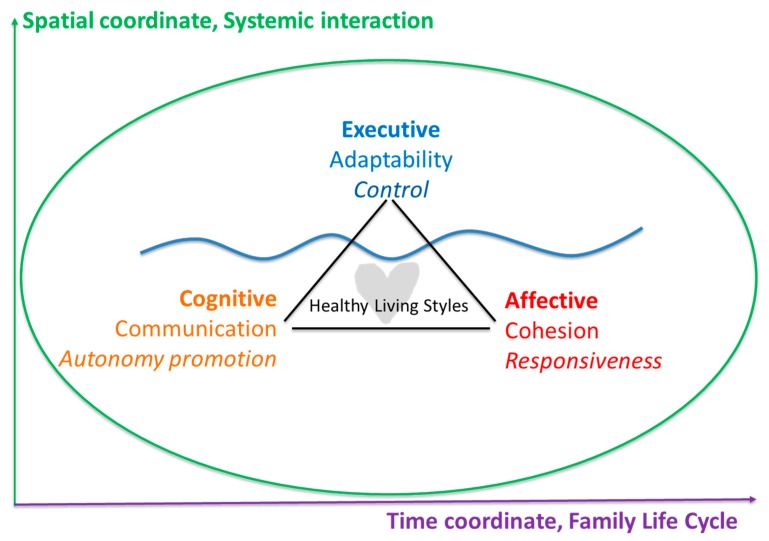
Proposal for a parenting style rooted in the three personal dimensions [30].

To categorize the healthy family functioning styles from a therapeutic perspective, Olson proposed the Circumplex model [30], which combines a behavioral variable, Adaptability, in establishing and maintaining norms, with an affective variable, Cohesion, which is related to closeness and warmth in relationships. He later added a third variable: Type of Communication—open or closed. Figure 1 shows the proposal to integrate the three variables mentioned in the following three personality dimensions: executive aspects related to the way of exercising control and establishing limits in the formation of the disposition, responsive acceptance of perceived cohesion in the Affective relationship, and type of communication that foments cognitive autonomy. The wavy line represents the horizon of the sea, which shows the observable aspect of the behavior while hiding the submerged part of the iceberg, which represent the cognitive–affective aspects. The time–space coordinates where our existence develops represent the vital dynamic aspect that requires constant adaptation.

In this scheme, the results of successive investigations can be incorporated based on the internal psychological mechanisms of the personality of both the parents and the children [31] and address the needs—both holistic and specific—of each family [32]. For example, according to the time–space characteristics of generational sensitivity and geographical area, the Control dimension can be operationalized at different levels—from parental monitoring, into consistent limits, and finally reaching severe discipline. The Responsiveness dimensions can be diversified in degrees of relatively structured cohesion, and the Communication type can be graduated through Socratic, inductive, imposing dialogue, etc. [33,34,35].

## 3. Advantages of the Tridimensional Proposal of Parenting Styles and Observations

The main advantage of the tridimensional style is its capacity to integrate the results of successive studies which lack consensus regarding terminology. The scheme of the personal psychological processes makes it possible to incorporate developmental–contextual variations without modifying the structure by calibrating the weight of each of the three dimensions: Control, Responsiveness, and type of Communication—or Adaptability, Cohesion, and Communication, according to the Olson model.

Until recently, the majority of studies pointed to the Authoritative style as the healthiest, but there has been an emerging tendency to nuance its efficacy [36,37,38,39] or substitute it with the Indulgent style in some countries in Europe and America, and in Iran [6,40,41,42]. These results do not involve modifying the recommended style, but rather maintaining the emotional quality in the parent/adolescent relationships and nuancing the family’s way of maintaining or establishing norms depending on its type of culture—individualist/collectivist and horizontal/vertical [43,44]. Research on Chinese families emigrating to the United States, the United Kingdom, and Japan has shown that this adaptation keeps the parents’ influence on their children from Evaporating [45,46]. The same phenomenon occurs in studies that highlight the importance of family cohesion [7,47] and the repeated recommendations about the need for emotional intelligence in order to correctly perform parenting tasks [48,49] in harmony with the emotional traits that characterize our society [50].

Likewise, the tridimensional style reflects the European Council’s Recommendation 2006/19, which highlights the importance of promoting the positive exercise of the parental role [51,52], as well as the conclusions of reviews on the Joint Construction models, which advise parents to lessen the Control as their children grow up and cede areas of decision-making to the adolescent’s autonomy in order to maintain both positive parenting and the children’s interior harmony [30,31,33,53].

On this point, it is important to re-dimension the parent–child discussions, understanding them as the other side of the coin of adolescents’ demand for greater autonomy, as a sort of transition ritual in our liquid culture [54]. In addition, the discussions that take place between the parents should not be underestimated, due to their negative effects on their children’s social adaptation, or their lack of coherence in observing consensual norms [52,55,56,57].

We have explained how various studies highlight the importance of family co-existence—the relational style—to the construction of a strong identity on which healthy lifestyles are built. Family coexistence extends to the design of a sustainable society, respectful of both unity and diversity [58,59]. It should be noted that parenting can also act as a risk factor for violent behaviors such as cyberbullying, active and proactive school violence among peers, hostility, and child-to-parent violence [60,61,62,63].

The aggressive nature of the current risk factors means that this task cannot be left only in the hands of families. Instead, it is necessary to articulate subsidiary systemic educational plans that reach where the family does not and involve the entire society, due to the inevitable fact that we all educate [64]. The good news is that some institutions have initiated successful experiments based on the results of these investigations. For example, *micro* actions have been taken in Minnesota, USA, whereby mentors of families were implemented in primary care medical centers, so far yielding good results [65]. From a macro perspective, there is also evidence of differential effects of family policies in countries such as South Korea, Finland, and Germany, compared to more individualistic policies in countries such as Sweden or Spain, which have had verified results in favor of family policies on international tests of educational achievement, like the Programme for International Student Assessment, PISA test [27].

The benefits of personal adjustment during adolescence translate into healthy lifestyles. These allow people to establish good relations with themselves, others and our common home, the biosphere, and contribute to human ecology and tackle the challenges of the 2030 Agenda of the United Nations for Sustainable Development [66,67].

## 4. Conclusions

1. An integrative and versatile parenting style is advisable to easily adapt to the changing traits of our liquid society.
2. Given that the affective dimension of family relationships acts as a protector factor in Authoritative or Indulgent profiles—which promote healthy adolescent lifestyles—in agreement with studies on adult bonding, it is important to promote emotional intelligence.
3. From a health perspective, there is a need to design efficient systemic educational plans that involve the whole society.
